# Advances in the Management of Aphakia

**DOI:** 10.1155/2022/9841758

**Published:** 2022-03-27

**Authors:** Georgios D. Panos, Craig Wilde, Paris Tranos, Zisis Gatzioufas

**Affiliations:** ^1^Department of Ophthalmology, Queen's Medical Centre, Nottingham University Hospitals, Nottingham, UK; ^2^Ophthalmica Eye Institute, Thessaloniki, Greece; ^3^Department of Ophthalmology, Basel University Hospitals, Basel, Switzerland

Aphakia is a condition in which the crystalline lens of the eye is not present in its normal position following surgical removal, perforating injury, congenital anomaly, or dislocation of the lens. It causes loss of accommodation, high hyperopia, and anisometropia.

The management of aphakia can be either conservative (spectacles or contact lenses) or surgical [1]. Surgical management of aphakia concerns both anterior and posterior segment surgeons and can be a real challenge, especially in paediatric patients where the visual system is still immature; because the child's eye continues to grow during childhood, certain complications are not acceptable [2–4].

In this Special Issue published in the *Journal of Ophthalmology*, Sidiropoulos et al. presented a new sutureless scleral fixation technique using a single-piece foldable acrylic Carlevale intraocular lens which they inserted in 27 eyes of 27 patients with poor capsular support [5]. The mean postoperative refraction at 6 months was −0.5 ± 0.99 *D*, while the postoperative complications were either resolved spontaneously or treated medically without the need for further surgery.

Massa and colleagues from the Geneva University Hospitals presented the SWISS IOL, a new minimally invasive technique for the scleral fixation of intraocular lenses (IOLs) in eyes without capsular support [6]. The postoperative spherical equivalent refraction ranged between −0.75 and −2.25, and no perioperative or postoperative complications were recorded while all IOLs were well centered postoperatively without any dislocation or tilt.

Finally, Karasavvidou and colleagues from the Nottingham University Hospitals provided a literature review on the surgical management of paediatric aphakia in the absence of sufficient capsular support presenting the advantages and disadvantages of each surgical technique [7].

*Georgios D. Panos* *Craig Wilde* *Paris Tranos* *Zisis Gatzioufas*

## References

[1] Wick R. E. (1979). Management of the aphakic patient. *Optometry and Vision Science*.

[2] Cheung C. S., VanderVeen D. K. (2019). Intraocular lens techniques in pediatric eyes with insufficient capsular support: complications and outcomes. *Seminars in Ophthalmology*.

[3] Gicquel J.-J., Langman M. E., Dua H. S. (2009). Iris claw lenses in aphakia. *British Journal of Ophthalmology*.

[4] Repka M. X. (2016). Visual rehabilitation in pediatric aphakia. *Developments in Ophthalmology*.

[5] Sidiropoulos G, Siskou E, Koronis S, Tranos P, Gatzioufas Z, Balidis M (2022). Novel sutureless scleral fixated IOL for inadequate or absent capsular support. *Journal of Ophthalmology*.

[6] Kecik M, Pajic B, Le Quoy O, Thumann G, Massa H (2021). The SWISS IOL technique (Small-Width incision scleral suture): a mini-invasive technique. *Journal of Ophthalmology*.

[7] Karasavvidou E.-M., Wilde C., Zaman A., Orr G., Kumudhan D., Panos G. D. (2021). Surgical management of paediatric aphakia in the absence of sufficient capsular support. *Journal of Ophthalmology*.

